# Post-cardiac injury syndrome: An evidence-based approach to diagnosis and treatment

**DOI:** 10.1016/j.ahjo.2021.100068

**Published:** 2021-11-19

**Authors:** Jahanzeb Malik, Syed Muhammad Jawad Zaidi, Abdul Sattar Rana, Ali Haider, Saleha Tahir

**Affiliations:** a Department of Cardiology, Rawalpindi Institute of Cardiology, Rawalpindi 46000, Pakistan; b Department of Medicine, Rawalpindi Medical University, Rawalpindi 46000, Pakistan; c Department of Respiratory Medicine, Shifa International Hospital, Islamabad 44000, Pakistan

**Keywords:** PCIS, Post-pericardiotomy syndrome, Post-traumatic pericarditis, Coronary artery disease

## Abstract

Post-cardiac injury syndrome (PCIS) is an umbrella term used for the post-pericardiotomy syndrome, post-myocardial infarction (MI) related pericarditis (Dressler syndrome), and post-traumatic pericarditis (percutaneous coronary intervention (PCI) or cardiac implantable electronic device (CIED) placement). All these conditions give rise to PCIS due to an inciting cardiac injury to pericardial or pleural mesothelial cells, leading to subsequent inflammation syndromes ranging from uncomplicated pericarditis to massive pleural effusion. We did a literature search on MEDLINE/PubMed for relevant studies using the terms “post-acute cardiac injury syndrome”, “post-cardiac injury syndrome”, “post-cardiotomy syndrome”, “post-pericardiotomy syndrome”, “post-MI pericarditis” and to summarize the body of evidence, all relevant full texts were selected and incorporated in a narrative fashion. Pathophysiology of PCIS is suggested as autoimmune-mediated in predisposed patients who develop anti-actin and anti-myosin antibodies following a cascade of cardiac injury in various forms. Colchicine and NSAIDs including ibuprofen are demonstrated as efficacious in preventing recurrent attacks of PCIS while corticosteroids show no benefit on prognosis and recurrence of the disease.

## 1 Introduction

Inflammation of the pericardium (pericarditis), in addition to pericardial effusion resulting from injury to the pericardium in termed post-cardiac injury syndrome (PCIS). It constitutes post-myocardial infarction (MI) related to pericarditis (Dressler syndrome), post-pericardiotomy syndrome, and post-traumatic pericarditis [1]. The term post-cardiotomy syndrome has been replaced with post-pericardiotomy syndrome as more evidence has demonstrated that the syndrome can occur after a minor injury to the pericardium, even when no other cardiac structures are manipulated. In addition, there is reason to believe that even a minor injury can provoke cardiac injury like percutaneous coronary interventions (PCI), cardiac implantable electronic device (CIED) placement, or radiofrequency ablation [2]. All these scenarios have a common inciting event of pericardial and/or pleural insult with subsequent pleuro-pericardial syndrome including pericarditis and pleural effusion [3].

The pericardium is a relatively avascular structure providing support to the heart, preventing excessive rocking movement in the mediastinum, and diastolic over dilation of the cardiac chambers [2]. Neuroreceptors located in the pericardium control heart rate and blood pressure during inspiration and the serous fluid contained in the pericardial sac provides frictionless movement of the heart in the thorax [4], [5]. The pericardium consists of fibrous (parietal) and serosal (visceral) layers derived from mesodermal germ cells after gastrulation into mesothelium in the first and second trimester [6]. The fibrous pericardium suspends the heart in the thorax attaching itself to the lungs, the cervical fascia, esophagus, adventitia of the coronary arteries, and the descending aorta [2].

PCIS was first described by Dressler in 1956 after acute MI [7]. While considered infrequent sequelae of pericardial insult, the actual incidence of PCIS is uncertain. This is because the available evidence is heterogeneous in post-MI patients. In the era before reperfusion, one study estimated the incidence of PCIS at 3% in acute MI patients, while another study found no such cases in their cohort [8]. Subsequently, in the era of primary PCI and emergent coronary artery bypass graft surgery (CABG), PCIS appears to have decreased in incidence. In a study of 201 patients with acute MI treated with thrombolysis, only one patient developed PCIS [9]. In another study of 743 patients with acute ST-elevation MI treated with PPCI, early post-MI pericarditis was recorded in 31 (4.2%) patients and late-onset pericarditis (Dressler syndrome) was demonstrated in only one patient [10]. However, PCIS has been reported in an increased number of cases after cardiac surgery, but the incidence is variable among different cohorts. In two investigations of 360 and 688 patients undergoing CABG, the authors reported an incidence of 15% and 9% of patients developing PCIS, respectively [11], [12]. Similarly, a large registry of 28,761 patients undergoing cardiothoracic surgery in Finland demonstrated a 1.7% incidence of PCIS [13]. In CIED implantation, PCIS is reported in less than 5% of the cases [14]. However, in one observational study of 4705 patients with CIED implantation, PCIS was reported in 0.1% of the patients [15]. PCI itself carries a 0.5% risk of pericardial complications and 1% risk for other percutaneous cardiac procedures [16], [17]. Table 1 highlights incidence of major cardiac injury syndromes.

**Table 1:** Type of presentations and incidence of PCIS. Post-cardiac injury syndrome (PCIS); myocardial infarction (MI); post-pericardiotomy syndrome (PPS); percutaneous coronary intervention (PCI); cardiac implantable electronic device (CIED).

PCIS	Incidence (%)
Post-MI	
Early (<7 days)	3%
Late (>7 days)	<1%
Post-cardiac surgery (PPS)	1%–15%
Percutaneous procedures	
PCI	0.5%
CIED implantation	<1% to 5%
Post-traumatic pericarditis	0.5% to 5%

In the 21st century, particularly in the developed countries, with the increasing age of the population in conjunction with newer indications for percutaneous cardiac interventions and cardiac surgery, there is an increased importance to understanding the mechanism and pathophysiology behind PCIS [2]. The objective of this review is to present up-to-date evidence on diagnosis, therapy, prognosis, and prevention of PCIS in a narrative layout.

## 2 Methods

Using MEDLINE/PubMed as the search engine, potentially relevant articles were extracted between 1956 and 2021. To summarize the body of evidence, all relevant full texts were selected and incorporated in a narrative fashion. The PubMed search was done with the terms “post-acute cardiac injury syndrome”, “post-cardiac injury syndrome”, “post-cardiotomy syndrome”, “post-pericardiotomy syndrome”, “post-MI pericarditis”. All conference proceedings and updated guidelines from the European Society of Cardiology and the American Heart Association were included in this review. All randomized controlled trials, observational studies, case reports, and reviews were searched for data extraction regarding state-of-the-art investigations and treatment of PCIS in 2021.

### 2.1 Pathophysiology

The pathophysiology of PCIS is not completely understood. In 1956, Dressler suggested a role of an auto-antigens-mediated hypersensitivity reaction after MI, leading to myocardial necrosis, and subsequent pericarditis and pleuropericardial effusions in predisposed patients [7]. After that, reports of PCIS after valve replacement surgeries and correction of congenital heart defects were published, which led to the revision of the etiopathogenesis of this phenomenon [18], [19].

**Fig. 1:**
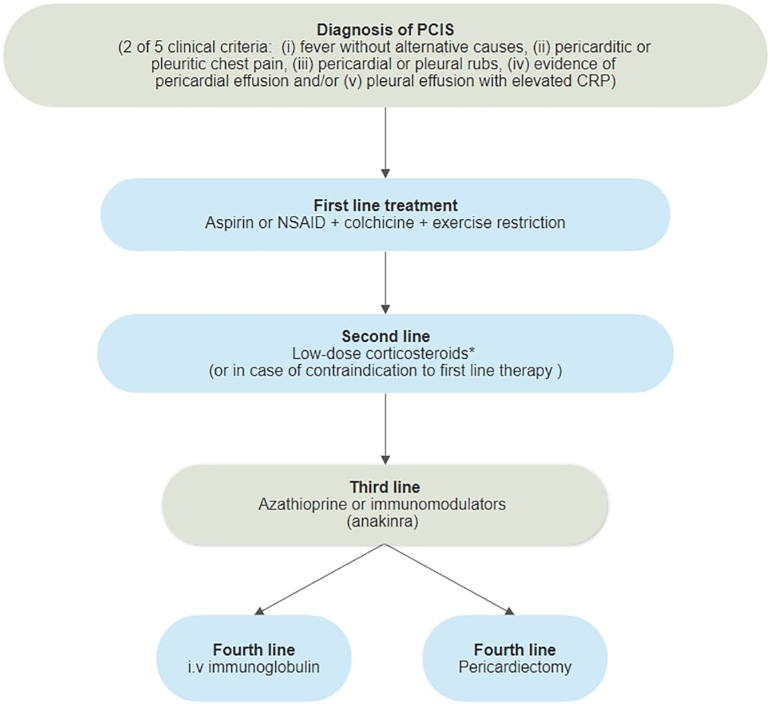
Pathogenesis of post-cardiac injury syndrome.

The current hypothesis of PCIS stands with autoimmune pathogenesis, inciting the cascade with damage to the pericardial and pleural mesothelial cells, caused by either necrosis of the myocardium, surgical trauma, blunt thoracic trauma, or iatrogenic damage to the pericardium (Fig. 1) [11], [20]. Although iatrogenic trauma during percutaneous cardiovascular interventions like PCI and CIED placement is regarded as minimal, the combination of this type of damage to the pleural or pericardial space can cause inflammatory cytokines to illicit a hyper-inflammatory state, leading to PCIS in predisposed individuals [21]. All invasive procedures involving the manipulation of vessels and cardiac structures have the potential for damaging the pericardium. Similarly, PCIS has been reported in blunt trauma (road traffic accidents), or sharp trauma (bullet or stab wounds) as well [22], [23].

The hypothesis of autoimmunity in PCIS is supported by the detection of anti-actin and anti-myosin antibodies in patients undergoing cardiac surgery in several investigations and a discrete latent period from cardiac injury to clinical onset of PCIS [24], [25]. The first study to identify the role of immune complexes in PCIS compared children in two groups after cardiac surgery [26]. Those who developed PCIS had increased levels of anti-myocardial antibodies resulting in myocardial injury. Subsequently, several prospective studies have demonstrated anti-actin and anti-myosin antibodies after cardiac surgical procedures in adults. However, the significance of these autoantibodies in pericardial and vascular injury is still unclear. An epiphenomenon of anti-heart antibodies was demonstrated in a prospective study of cardiac surgery patients (n = 20) [27]. Repeated sampling for anti-heart antibodies was carried out before the procedure, and after several weeks' post-procedure. All were seronegative before the procedure. Out of 20 patients, three developed PCIS and became seropositive within two weeks. One other registry illustrated a difference of causality for early (within seven days) versus late effusions in suspected PCIS [28]. Anticoagulant therapy was demonstrated as a contributing factor in most of the early effusions while one-third of late effusions were caused by PCIS.

Furthermore, the role of perioperative viral infections is being debated in the pathophysiology of PCIS. There is a reported seasonal variation in PCIS along with elevated viral titers and anti-heart antibodies, suggesting that viral infections may have causality with PCIS [3]. However, one investigation with orthotopic cardiac transplant also described PCIS in immunosuppressed children, suggesting that this syndrome is not always an autoimmune process [29]. Further studies are needed to develop a model for the mechanism of action of PCIS and its pathogenesis.

### 2.2 Clinical presentation and investigations

In PCIS, there is a clinical overlap of signs and symptoms with acute pericarditis or pleural effusion in the setting of other clinical scenarios. The most important features of PCIS are the predisposition to cardiac injury in terms of prior invasion of the pericardium, myocardium, or manipulation of the pleural cavity [11]. Furthermore, there is a latency period of weeks to months between the inciting injury and the presentation of pleural or pericardial effusion. This latent period is highly variable among patients. However, the presentation and clinical course of PCIS are comparable to the acute sequelae of cardiac injury [8], [16]. Table 2 shows diagnostic criteria for PCIS.

**Table 2:** Diagnostic criteria of PCIS. Post-cardiac injury syndrome (PCIS); C-reactive protein (CRP).

	Diagnosis of PCIS^a^
(i)	Fever without alternative cause
(ii)	Pleuritic or pericarditic chest pain
(iii)	Pleural or pericardial rub
(iv)	Evidence of pericardial effusion
(v)	Pleural effusion with raised CRP

a 2015 ESC Guidelines for the diagnosis and management of pericardial diseases: The Task Force for the Diagnosis and Management of Pericardial Diseases of the European Society of Cardiology (ESC) endorsed by: The European Association for Cardio-Thoracic Surgery (EACTS).

In a registry of 360 patients, 54 (15%) developed PCIS [11]. The majority of the patients developed symptoms of PCIS in the first month (79.6%), 13% in the second, and 7.4% in the third month. Specific symptoms and signs included pleural effusion (92.6%), pericardial effusion (88.9%), elevated markers of inflammation (74.1%), pleuritic chest pain (55.6%), fever (53.7%), and pericardial rub (32.3%). Chylothorax and/or hydropericardium is a rare complication of PCIS which occasionally occurs in children treated with extensive surgical interventions for complex congenital heart diseases [30].

There are no standard diagnostic criteria for PCIS and mostly it is a diagnosis of exclusion. Initial investigations include inflammatory markers like erythrocyte sedimentation rate (ESR), C-reactive protein (CRP), troponin T or I, and complete blood count (CBC). In more than 83% of the patients, these markers are elevated in acute pericarditis as well as PCIS [31]. In the absence of other diseases, these inflammatory markers can help in suspecting PCIS, and a surrogate for measuring response to anti-inflammatory treatment. Troponin T or I levels are increased if there is the involvement of epicardium or myocardial injury in acute settings. Hence, their specificity in the setting of acute MI is low [32], [33]. However, in late presentations, it can point towards Dressler's syndrome, therefore, making it an important marker later in the disease. The availability of high-sensitivity cardiac troponin T (hs-cTnT) has increased sensitivity when compared with conventional troponins in ACS. However, non-ACS cause cannot be ruled out and hence, this marker is not used for PCIS. A chest X-ray can show an enlarged cardiac shadow in the setting of pericardial effusion or tamponade and help in excluding an alternative diagnosis of chest pain or dyspnea [34]. Other causes like malignancy, uremia, connective tissue disorders should also be kept in mind when diagnosing PCIS. These common causes should be ruled out first.

Electrocardiogram (ECG) is the single most important basic investigation in diagnosing active pericarditis from PCIS. ECG changes occur in 20% of acute pericarditis cases, presenting as diffuse ST-segment depression and PR segment elevation (Stage 1), normalization of ST and PR segments with relative flattening of T-waves (Stage 2), T-wave inversions (Stage 3), and finally normalization of ECG (90%) or persistent T-wave inversions (10%–12%) (Stage 4) [34]. These stages are usually absent in PCIS and present as nonspecific ST-T changes or pseudo-infarct patterns. Transthoracic echocardiography (TTE) is another readily available diagnostic technique that can be performed at the bedside for assessment of cardiac anatomy, hemodynamics, and function. In patients presenting with pericardial effusion, TTE can provide accurate quantification for guidance during pericardiocentesis [35], [36]. A limitation of TTE is that anatomy of the pericardium is not visualized very well (Table 3).

**Table 3:** Drug regimen and follow-up for post-cardiac injury syndrome.

Drug	Anti-inflammatory dose	Duration of initiating or maintenance dose	Tapering regimen	Follow-up
First-line therapy				• Clinic follow-up with inflammatory markers at 4 weeks post diagnosis • If symptomatic or inflammatory markers remain elevated, close clinic follow-up every 2 weeks • If symptoms resolve and inflammatory markers normalize, clinic follow-up and echocardiography at 3 months
Aspirin	750–1000 mg q6h	3–4 weeks	Decrease dose by 250 mg per week	
**or**				
Ibuprofen	600–800 mg q6h	3–4 weeks	Decreased dose by 200 mg per week	
**or**				
Indomethacin	25–50 mg q8h	3–4 weeks	Decrease dose by 25 mg per week	
**plus**				
Colchicine	0.6 mg q12h	3 months (first episode) 6 months (recurrent)	Not tapered	
Second-line therapy (for refractory cases or contraindication to NSAIDs)				
Prednisolone	0.2–0.5 mg/kg q24h	2–4 weeks	Gradual tapering by 0.2 mg/kg per week	
**plus**				
Colchicine	0.6 mg q12h	3–6 months	Not tapered	
Third-line therapy (or pericardiectomy)				
Anakinra	1–2 mg/kg q24h	Several months	Slowly taper over 3 months	
Azathioprine	1–3 mg/kg q24h	Several months	Not tapered	
IVIG	400–500 mg/kg q24h	5 days	Not tapered	

Advanced cardiac imaging modalities include computed tomography (CT) and cardiac magnetic resonance (CMR) for visualizing intricate structures in the heart. They provide detailed information about pericardium and its underlying structures, pathology, and functions. They can detect underlying inflammatory processes and differentiate between acute and chronic pericarditis [37], [38], [39]. However, both imaging modalities have a limited role in hemodynamically unstable patients, renal dysfunction, pregnancy, and arrhythmias as it requires prolonged supine position along with breath-hold sequences. Additionally, it exposes patients to ionizing radiations [40].

### 2.3 Treatment and prevention

**Fig. 2:**
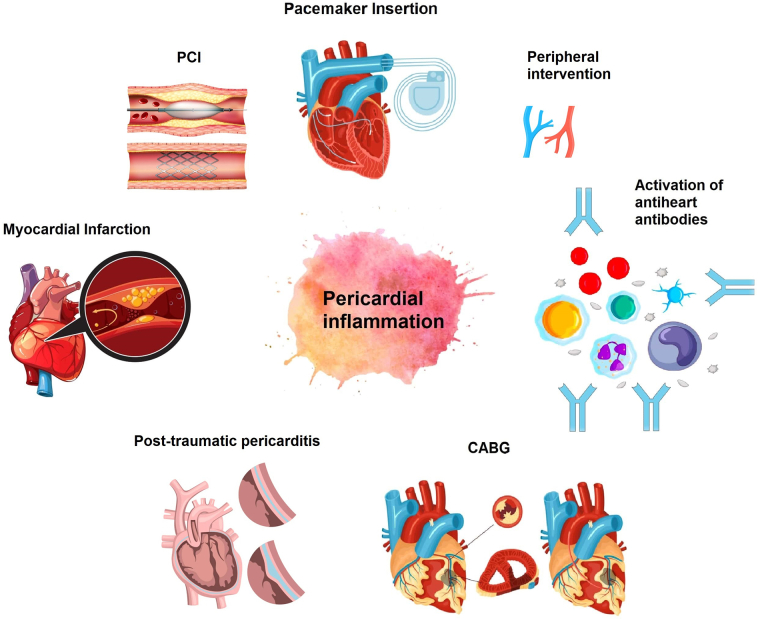
Therapeutic algorithm for treatment of post-cardiac injury syndrome. *Rule out infection, malignancy, connective tissue disorder, uremia, and other causes first.

The treatment algorithm for PCIS is the same as recommended in 2015 European Society of Cardiology (ESC) guidelines for the diagnosis and management of pericardial diseases [34]. An algorithm is illustrated in Fig. 2. The main goal of therapy for PCIS is to decrease pericardial inflammation for symptomatic improvement of the patients until the disease state subsides. Any clinical presentation with at least one predictor of poor prognosis (fever > 38 °C, symptoms over several days, large pericardial effusion > 20 mm, and NSAID non-responder) should be managed in the hospital. The most important non-pharmacologic recommendation is to restrict intense physical activity for at least three months up to six months (in athletes) or after symptoms have subsided and inflammatory markers (ESR, CRP, ECG, TTE) are normalized (both athletes and non-athletes) [34].

NSAIDs are the first-line treatment strategy in PCIS, and owing to the dual anti-inflammatory and anti-platelet effect of aspirin, it is the NSAID of choice in patients with acute on concomitant ischemic heart disease (IHD). In PCIS, aspirin is given at a dose of 750–1000 mg every 8 h, and indomethacin is administered at 600 mg every 8 h for weeks to months until symptom resolution or normalization of the inflammatory markers. The dose should be tapered by 200–400 mg over a period of two weeks. Colchicine has demonstrated potent anti-inflammatory effects in IHD [34]. Major multicenter trials have exhibited reduced all-cause and cardiovascular mortality in patients with the acute coronary syndrome (ACS) [41]. Similarly, colchicine is recommended in MI-associated pericarditis and PCIS [34]. In patients with failure or contraindication to NSAIDs, it should be advised at a dose of 0.5 mg 12 h for weeks to months and no tapering is mandatory for stopping after symptom resolution. For resistant or recurrent pericarditis, corticosteroids (prednisolone 0.2–0.5 mg/kg/day) should be administered as second-line agents [42]. However, the use of corticosteroids should be limited to severe non-responders to NSAIDs because steroids favor chronicity, and have myriad of side effects [43]. Immunomodulators (anakinra, azathioprine) are recommended for corticosteroid-dependent PCIS unresponsive to colchicine. Similarly, as a last resort, pericardiectomy may be considered in select patients, if medical therapy fails [44], [45].

There are several investigations comparing treatment options for PCIS. In colchicine for prevention of the post-pericardiotomy syndrome and postoperative atrial fibrillation (COPPS-2) trial published in 2014 enrolled 360 patients for cardiac surgery [46]. Colchicine reduced the incidence of PCIS by half when compared with the placebo group (9% vs. 21%; p = 0.002) as a primary outcome. In contrast, two trials demonstrated no efficacy of aspirin and corticosteroids in preventing PCIS [47], [48]. However, more studies are needed to assess the outcomes of aspirin in reducing the incidence of PCIS in patients after PCI or pacemaker implantation, and valvular surgery.

Usually, the prognosis of PCIS is good for most of the patients, a recurrence rate of 10% to 15% has been reported [49]. Furthermore, there is a 2% risk of developing constrictive pericarditis, hence a longer follow-up for several years should be recommended to all patients with PCIS [50].

## 3 Conclusion

PCIS includes pleuro-pericardial inflammation and effusion syndromes after traumatic mesothelial cell injury to the pericardium or pleural cavity. Several triggers have been identified in this review that can cause PCIS, including PCI, CIED implantation, and cardiac surgical procedures. PCIS can present as uncomplicated pericarditis to the complex case of pleuro-pericarditis, tamponade, or massive pleural effusion. Mechanism of action behind PCIS seems to be immune-mediated and certain individuals with a predisposition to the disease. The treatment strategy is based on an anti-inflammatory regime with NSAIDs, colchicine, and restricted activity as the first-line management. In addition, colchicine is reported to be safe and efficacious in preventing recurrence of PCIS. However, relapse of the disease in a small percentage of individuals is possible with progression to constrictive pericarditis. The overall prognosis of PCIS is benign and reassurance is important to the individuals affected with this syndrome, a recurrence rate of 10% to 15% has been reported. In addition, because of a small risk of developing constrictive pericarditis, longer-term follow-up over several years is warranted.

## Funding

Author received no specific funding for this article.

## CRediT authorship contribution statement

JM = concept, design, methodology, writing-first & final draft; SMJZ = first draft, literature search, methodology; ASR = first draft; AH = first and final draft, images, tables; ST = first draft.

## Declaration of competing interest

Author declares no competing interest.
